# Seasonal variation in vitamin D status, bone health and athletic performance in competitive university student athletes: a longitudinal study

**DOI:** 10.1017/jns.2020.1

**Published:** 2020-02-10

**Authors:** Saskia L. Wilson-Barnes, Julie E. A. Hunt, Emma L. Williams, Sarah J. Allison, James J. Wild, Joe Wainwright, Susan A. Lanham-New, Ralph J. F. Manders

**Affiliations:** 1 Department of Nutritional Sciences, School of Biosciences and Medicine, Faculty of Health and Medical Sciences, University of Surrey, Guildford, Surrey GU2 7XH, UK; 2 Division of Women's, Children's and Clinical Support, Imperial College Healthcare NHS Trust, London W12 0NN, UK

**Keywords:** Vitamin D, University athletes, Bone, Muscle strength, Athletic performance, Physiology, AF, aerobic fitness, PTH, parathyroid hormone

## Abstract

Vitamin D deficiency has been commonly reported in elite athletes, but the vitamin D status of UK university athletes in different training environments remains unknown. The present study aimed to determine any seasonal changes in vitamin D status among indoor and outdoor athletes, and whether there was any relationship between vitamin D status and indices of physical performance and bone health. A group of forty-seven university athletes (indoor *n* 22, outdoor *n* 25) were tested during autumn and spring for serum vitamin D status, bone health and physical performance parameters. Blood samples were analysed for serum 25-hydroxyvitamin D (s-25(OH)D) status. Peak isometric knee extensor torque using an isokinetic dynamometer and jump height was assessed using an Optojump. Aerobic capacity was estimated using the Yo-Yo intermittent recovery test. Peripheral quantitative computed tomography scans measured radial bone mineral density. Statistical analyses were performed using appropriate parametric/non-parametric testing depending on the normality of the data. s-25(OH)D significantly fell between autumn (52·8 (sd 22·0) nmol/l) and spring (31·0 (sd 16·5) nmol/l; *P* < 0·001). In spring, 34 % of participants were considered to be vitamin D deficient (<25 nmol/l) according to the revised 2016 UK guidelines. These data suggest that UK university athletes are at risk of vitamin D deficiency. Thus, further research is warranted to investigate the concomitant effects of low vitamin D status on health and performance outcomes in university athletes residing at northern latitudes.

Interest in vitamin D has risen significantly over recent years due to its numerous roles in human health and its potential role in athletic performance^(1)^. A sufficient vitamin D status (>50 nmol/) is most commonly associated with skeletal health and the prevention of osteoporosis^(2)^. The role of vitamin D in muscular strength and power is currently under much speculation. There is evidence to suggest that vitamin D can increase the sensitisation of Ca binding sites at the sarcoplasmic reticulum^(3)^ and influence muscle growth and differentiation; in particular muscle type II fibres^(4)^. Due to the presence of vitamin D receptors within cardiac muscle and vascular tissues, it is proposed that 1,25 dihydroxyvitamin D (1,25(OH)_2_D) may have a positive influence upon VO_2max_^(5,6)^. However, research into the use of vitamin D as an ergogenic aid has demonstrated no improvements in muscular function^(7)^ unless the participants presented with deficiency (<30 nmol/l) (1) or severe deficiency (<12·5 nmol/l)^(8)^. Due to the large heterogeneity of studies upon the aspects of physical performance, the ergogenic effects of vitamin D remain unclear^(9)^. It is well established that vitamin D has a significant influence upon bone health and mineralisation^(10)^, although research examining vitamin D and its association with bone mineral density did not find any associations in athletic populations^(4,6)^. There is, however, emerging evidence to suggest that a low vitamin D status is associated with an increased risk of stress fractures in military recruits residing within the UK and Finland (these studies utilised <50 and <75 nmol/l as their cut-off values for low vitamin D status, respectively)^(11,12)^. Given that university-level athletes residing in the UK are likely to have low vitamin D status, further investigation into their bone health is warranted.

As UVB exposure is a primary source of vitamin D^(13)^, latitude plays a vital role in vitamin D status and individuals living above or below 40° of the equator are at risk of low vitamin D status. Therefore, the UK population is unable to synthesise vitamin D from mid-October to the beginning of April^(14)^. Recent evidence has emerged that physically active populations are at risk of developing vitamin D insufficiency (<50 nmol/l)^(1)^, similar to the UK population^(15)^. One potential reason for this risk is that many highly active individuals and athletes spend large amounts of time indoors competing and training and therefore receive limited year-round sunlight exposure^(13,14)^. This is supported by evidence from studies conducted at different latitudes, including Australia^(16)^, the USA^(17,18)^, Europe^(19,20)^ and the UK^(21,22)^. An Australian study^(16)^ found that their elite indoor athletes had significantly lower vitamin D status when contrasted to their outdoor groups (90 *v.* 131 nmol/l, respectively), suggesting that indoor athletes may be at increased susceptibility to vitamin D deficiency in winter months due to a lack of subcutaneous exposure to sunlight.

Although there is evidence relating to elite-level athletes, who receive adequate nutrition support from a sport nutritionist or dietitian, university-level/amateur athletes rarely receive any nutritional support. There is also a lack of data on UK university-level athletes; thus vitamin D deficiency may be more prevalent in this population. Subsequently, the purpose of the present study was therefore to examine the prevalence of vitamin D deficiency in university-level athletes throughout a competitive season. A secondary aim was to evaluate the associations between vitamin D status on markers of bone health and physical performance in indoor and outdoor athletes. We hypothesised that university-level athletes would be vitamin D insufficient (<50 nmol/l) in the spring term, with an adverse effect upon physical performance and predictors of bone health.

## Methods

### Participants

A total of forty-seven university-level athletes attending the University of Surrey (51·2°N) were recruited into the study. Participants were included if they trained for ≥4 h/week and competed in the national British Universities and Colleges Sports (BUCS) competition. Exclusion criteria were: BMI <18 kg/m^2^, the regular use of sunbeds or sun holidays between October and February, the regular consumption of supplements containing vitamin D or pregnancy. Written informed consent was obtained from participants. The present study was conducted according to the guidelines laid down in the Declaration of Helsinki and all procedures involving human subjects were approved by the University of Surrey Ethical Committee.

### Study design

**Fig. 1.**
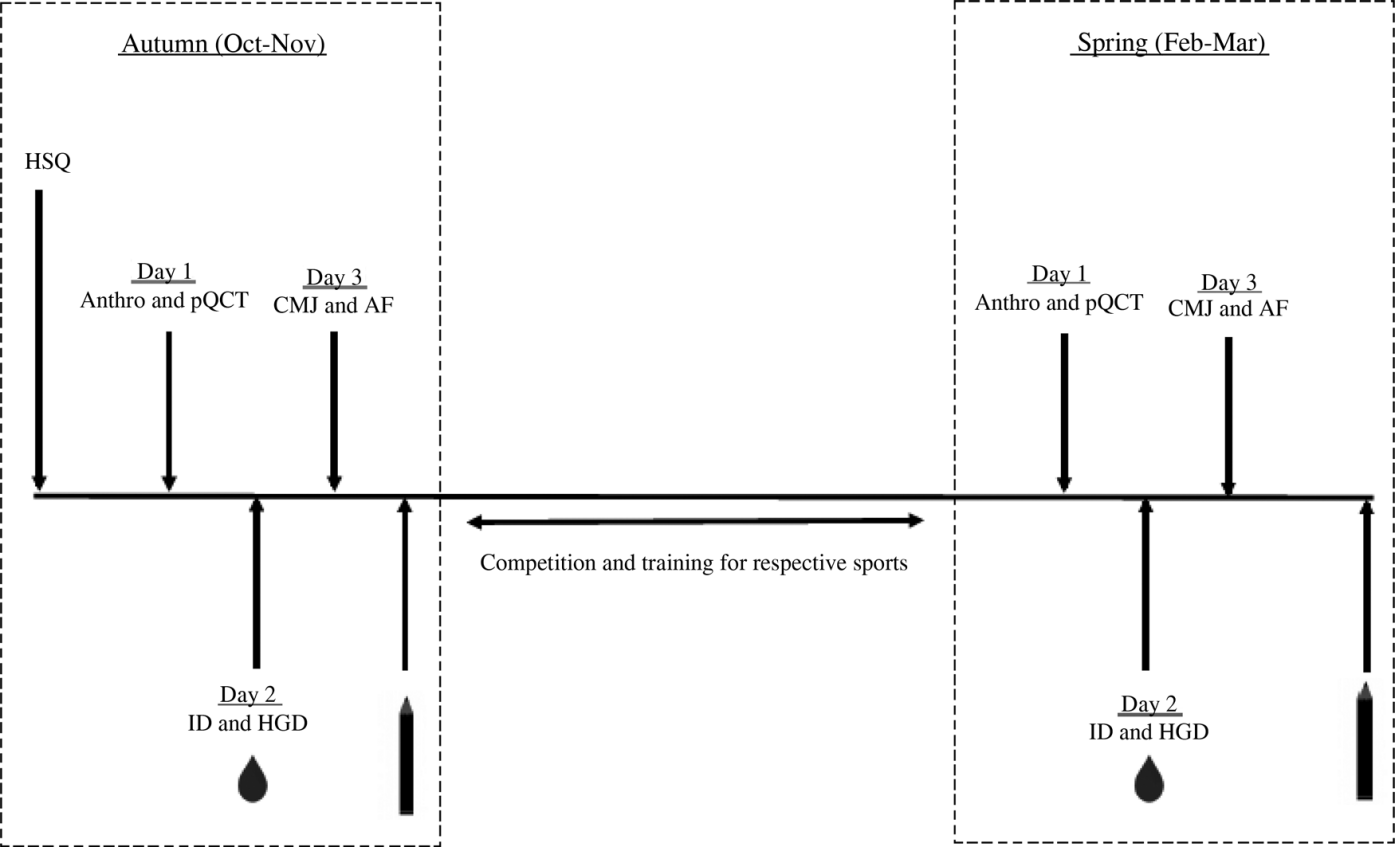
Schematic diagram of study design. HSQ, health screening questionnaire; Anthro, anthropometrics; pQCT, peripheral quantitative computed tomography of non-dominant radius; CMJ, counter movement jump; AF, aerobic fitness; ID, isokinetic dynamomotry; HGD, handgrip dynamomotry; 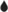, serum and plasma samples collected (16 ml) and finger prick test; 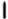, 5-d self-reported food diary collected.

This longitudinal observational study assessed vitamin D status throughout a competitive British Universities and Colleges Sports (BUCS) season and assessed dietary intake, bone health and physical performance in autumn (October/November 2015) and spring (February/March 2016). At baseline participants were requested to complete a health-screening questionnaire to assess their suitability for inclusion into the study. This included vitamin D-specific questions such as self-reported ethnicity, sun holidays, the daily intake of nutritional supplements and the regular use of sunbeds. Autumn and spring test periods consisted of three separate test days at baseline and post-observation (see Fig. 1 for a study overview). Dietary intake was measured using a 5-d food diary; participants were instructed to include at least one weekend day. Diaries were analysed by the researcher using DietPlan6 (Forestfield Software Ltd).

### Anthropometric measurements

Height was measured using a fixed stadiometer, and body mass and composition were obtained using a Tanita Body Composition Analyser (MC-180MA; Tanita Cooperatives) whilst participants stood on the device barefoot and wearing light clothing.

### Peripheral quantitative computed tomography

A peripheral quantitative computed tomography (pQCT) scan (XCT 2000L; Stratec Medizintechnik GmbH) was performed on the non-dominant radius to measure volumetric bone mineral density across the distal end (4 %) and mid-shaft (66 %). Radial bone composition and density were assessed within this study as the weight-bearing nature of particular sports may confound the results when assessing the tibia. Radial length was determined as the distance (mm) from the styloid process to the olecranon. The non-dominant arm was identified by self-report from the participant.

### Vitamin D and parathyroid hormone measurement

Participants were instructed to visit the laboratory between 07.30 and 11.00 hours after an overnight fast (≥8 h). Blood samples were collected into an EDTA tube (6 ml) and centrifuged at 1300 ***g*** and 4°C for 10 min. Serum blood (10 ml) was kept at room temperature for 1 h before centrifugation at 1300 ***g*** and 22°C. Samples of plasma and serum were immediately frozen and stored at −20°C until analyses. Serum vitamin D status was assessed using liquid chromatography–tandem MS (LC-MS/MS) on a Waters Acuity TQD using a pentafluorophenyl (PFP) column following supported liquid extraction. Plasma parathyroid hormone (PTH) was measured using intact PTH assays (Abbott Laboratories). Both measurements were undertaken in an accredited laboratory.

### Muscle strength and jump height

Isometric knee extensor strength was assessed on an isokinetic dynamometer (CSMI Humac Norm). Participants completed a 5-min warm up on a cycle ergometer (about 75 W) before being seated on the dynamometer with their non-dominant leg (also self-reported by the participant) secured at 90° knee flexion. Participants performed three 5-s maximal contractions separated by a 1-min rest to determine knee extensor strength. Handgrip dynamometry was measured for the non-dominant arm, using a Takei Digital dynamometer (5401; Takei Scientific Instruments Co.). The test was repeated three times consecutively. Participants completed three counter movement jumps (Optojump; Microgate Co.) with hands on hips, separated by a 2-min rest. The peak results for isometric strength, handgrip and counter movement jumps of the three were used for further analysis.

### Aerobic fitness

Aerobic fitness (AF) was measured using a standardised Yo-Yo Intermittent Recovery Test Level 1^(23)^. Participants performed two 20 m shuttle runs at increasing speeds, interspersed with 10 s periods of active recovery, controlled by signals from an audio device. Participants ran until voluntary exhaustion or were instructed to stop the test when they were unable to maintain the speed (failed to meet the line in synchronisation with the audio signal on two separate occasions). The distance covered at that point was recorded as the test result.

### Statistical analysis

All statistical analysis was performed using SPSS software (version 24; SPSS Inc.). Data were checked for normality using the Shapiro–Wilkinson test. Paired and independent *t* tests or the non-parametric equivalent were conducted on this dataset. Associations between variables were examined using Pearson or Spearman correlation coefficients. To investigate the potential adverse effects of vitamin D deficiency on markers of physical performance, subjects were divided into the following vitamin D status categories according to their serum 25-hydroxyvitamin D status: vitamin D ‘deficiency’ (<25 nmol/l); vitamin D ‘insufficiency’ (25–50 nmol/l); vitamin D ‘sufficiency’ (50–75 nmol/l) and vitamin D ‘above sufficiency’ (>75 nmol/l). Statistical analysis of these categories was undertaken using a one-way ANOVA test (or the non-parametric equivalent) with *post hoc* comparisons using the Tukey honestly significant difference test. Significance was set at *P* ≤ 0·05.

## Results

### Participant characteristics

A group of forty-seven participants (sixteen women and thirty-one men) were tested in autumn and spring (Table 1). Athletes competed in a range of sports: those who participated in basketball, cheerleading, mixed martial arts, rowing, squash, swimming and table tennis (*n* 22) were classified as indoor athletes. Athletes who participated in athletics, American football, football, hockey, lacrosse, rugby, triathlon and ultimate frisbee (*n* 25) were classified as outdoor athletes. Data from the health screening questionnaires revealed that our athletes were of White–Caucasian (*n* 39), Asian–Indian (*n* 6) and Asian–Chinese (*n* 2) descent.

**Table 1.** Participant characteristics and physical parameters (Mean values and standard deviations)

	Autumn				Spring			
	Outdoor (*n* 22)		Indoor (*n* 25)		Outdoor (*n* 22)		Indoor (*n* 25)	
	Mean	sd	Mean	sd	Mean	sd	Mean	sd
Age (years)‡	21·0	1·8	20·0	1·4	–	–	–	–
BMI (kg/m^2^)	25·4	3·4	23·3*	2·7	25·7	3·8	23·5†	2·9
Body fat (%)	19·7	6·9	19·5	6·4	20·6	6·5	20·1*	6·5
FFM (kg)	64·8	9·5	58·4*	12·2	63·5	10·6	57·5*	10·9
Training (h)‡§	5·8	3·3	7·3*	2·9	5·2	2·4	7·14	4·2
Vitamin D intake (μg/d)‡§	3·3	2·5	2·2	2·0	4·4	5·0	2·4	1·6
Ca intake (mg/d)‡	1022·4	370·6	932·9	493·7	814·2	198·0	678·3*	233·5
25(OH)D status (nmol/l)	54·3	25·3	57·7	22·0	31·0	17·5	31·0	16·1
PTH (pmol/l)‡§	6·6	3·2	6·5	4·3	5·5	2·2	7·0	5·1
Knee extensor strength (Nm)	259·5	51·8	245·1*	99·0	276·4	71·2	234·8†	61·8
Handgrip (kg)	42·0	10·3	37·0	10·6	41·2	11·6	36·9	10·2
CMJ (cm)‡	31·8	8·4	29·5	9·4	33·7	7·3	32·2	7·6
VO_2max_ (ml/kg per min)‡	42·9	3·2	43·1	2·8	43·7	2·2	42·5	3·2

FFM, fat-free mass; Ca intake, Ca intake from self-reported 5-d food diary; 25(OH)D, 25-hydroxyvitamin D; PTH, parathyroid hormone; CMJ, counter movement jump; VO_2max_, aerobic fitness.

* Mean value is significantly different from that for outdoor athletes in the same season (*P* < 0·05; independent *t* test/Mann–Whitney *U* test between indoor and outdoor athletes).

† Mean value is significantly different from that for the same type of athlete in autumn (*P* < 0·05; paired *t* test/Wilcoxon rank test).

‡ Not normally distributed in autumn.

§ Not normally distributed in spring.

### Dietary intake

Dietary analysis illustrated that participants did not meet current UK dietary reference intake (10 µg/d)^(24)^ for vitamin D with a menial intake of 2·7 (sd 2·3) and 3·2 (sd 3·3) µg/d during autumn and spring, respectively. However, Pearson correlations revealed no association between vitamin D status and intake. Ca intake was adequate during the winter term for both athlete groups as they exceeded the current recommendations of 700 mg/d^(25)^. However, during the spring term there was a significant decline in Ca consumption for the outdoor (814·2 (sd 198·0) mg/d) and indoor (678·3 (sd 233·5) mg/d) groups (*P* = 0·031).

### Physical performance

Aspects of physical performance did not improve with training and competition between seasonal time points (Table 1). Indoor athletes' self-reported training hours were consistently higher than their outdoor counterparts in autumn (7·3 (sd 3·0) *v.* 5·8 (sd 3·3) h) and spring (7·1 (sd 4·2) *v.* 5·2 (sd 2·4) h) (*P* = 0·018). There were no differences between indoor and outdoor athletes exhibited in AF during the autumn (*P* = 0·614) and spring (*P* = 0·128) terms. Peak knee extensor isometric strength was higher in the outdoor athletes during both the spring (*P* = 0·023) and the autumn (*P* = 0·044). Handgrip strength did not differ between outdoor athletes during both seasons (*P* = 0·135 and 0·186). There were no changes observed between seasons for jump height.

When controlling for height, weight and fat-free mass there was a positive association between isometric knee strength and AF in all athletes during the autumn term (*r* 0·378, *P* = 0·008; *r* 0·391, *P* = 0·011, respectively). This was not observed during the spring. Handgrip strength and vitamin D status (*r* 0·332; *P* = 0·039) were positively associated during spring for the entire cohort. There was a positive association for the outdoor group between vitamin D status, AF (*r* 0·563; *P* = 0·019) and counter movement jumps (*r* 0·544; *P* = 0·024) during the autumn measurement only. None of the physical performance parameters was associated with vitamin D status during both seasons in the indoor group.

### Bone health indices

Indoor athletes exhibited a significantly lower cortical area and trabecular density compared with their outdoor counterparts in autumn (Table 2), although it did not approach significance during spring. The indoor group elicited a lowered bone mineral content and density at both time points at the distal (4 %) and proximal (66 %) radius. Bone mineral content and density at either distal or proximal sites were not associated with vitamin D status within the combined group. However, vitamin D was associated with total area at the proximal site in the indoor group during spring (*r* 0·532; *P* = 0·007). When correcting for height and weight, results were no longer significant (*r* 0·336; *P* = 0·126). In addition to this cortical density increased significantly in spring by 20·2 mg/cm^3^ in the combined group from autumn to spring (*P* = 0·010).

**Table 2.** Bone parameters (Mean values and standard deviations)

	Autumn				Spring			
	Outdoor (*n* 22)		Indoor (*n* 25)		Outdoor (*n* 22)		Indoor (*n* 25)	
	Mean	sd	Mean	sd	Mean	sd	Mean	sd
4 % Radius								
BMC (g/cm)	1·51	0·3	1·30	0·3	1·50	0·3	1·34	0·3
Total CSA (mm^2^)‡	411·6	82·5	403·0	115·3	406·5	81·4	397·9	96·0
BMD (mm^3^)	375·1	59·0	331·0	55·4	381·2	53·9	339·4	53·5
Trab vBMD (mg/cm^3^)†	255·5	76·1	228·4*	57·8	253·5	54·9	221·4	52·8
66 % Radius								
BMC (g/cm)†‡	1·3	0·5	1·2	0·4	1·5	0·7	1·5	0·8
Total CSA (mm^2^)†‡	215·8	107·0	188·9	111·2	249·3	140·1	250·6	180·6
BMD (mm^3^)‡	629·7	94·2	638·6	130·6	610·0	162·7	663·2	91·0
Crt CSA (mm^2^)†‡	80·8	29·5	79·4*	29·0	109·2	52·2	92·0	33·6
Crt vBMD(mg/cm^3^)†‡	1044·1	63·1	1055·8	74·2	1065·8	38·4	1074·9	61·9
SSI (mm^2^)†‡	414·7	379·4	315·6	173·1	529·0	594·4	432·9	521·8

BMC, bone mineral content; CSA, cross-sectional area; BMD, bone mineral density; Trab, trabecular; vBMD, volumetric bone mineral density; Crt, cortical; SSI, strength strain index.

* Mean value is significantly different from that for outdoor athletes in the same season (*P* < 0·05; independent *t* test/Mann–Whitney *U* test between indoor and outdoor athletes).

† Not normally distributed in autumn.

‡ Not normally distributed in spring.

### Vitamin D status and parathyroid hormone

**Fig. 2.**
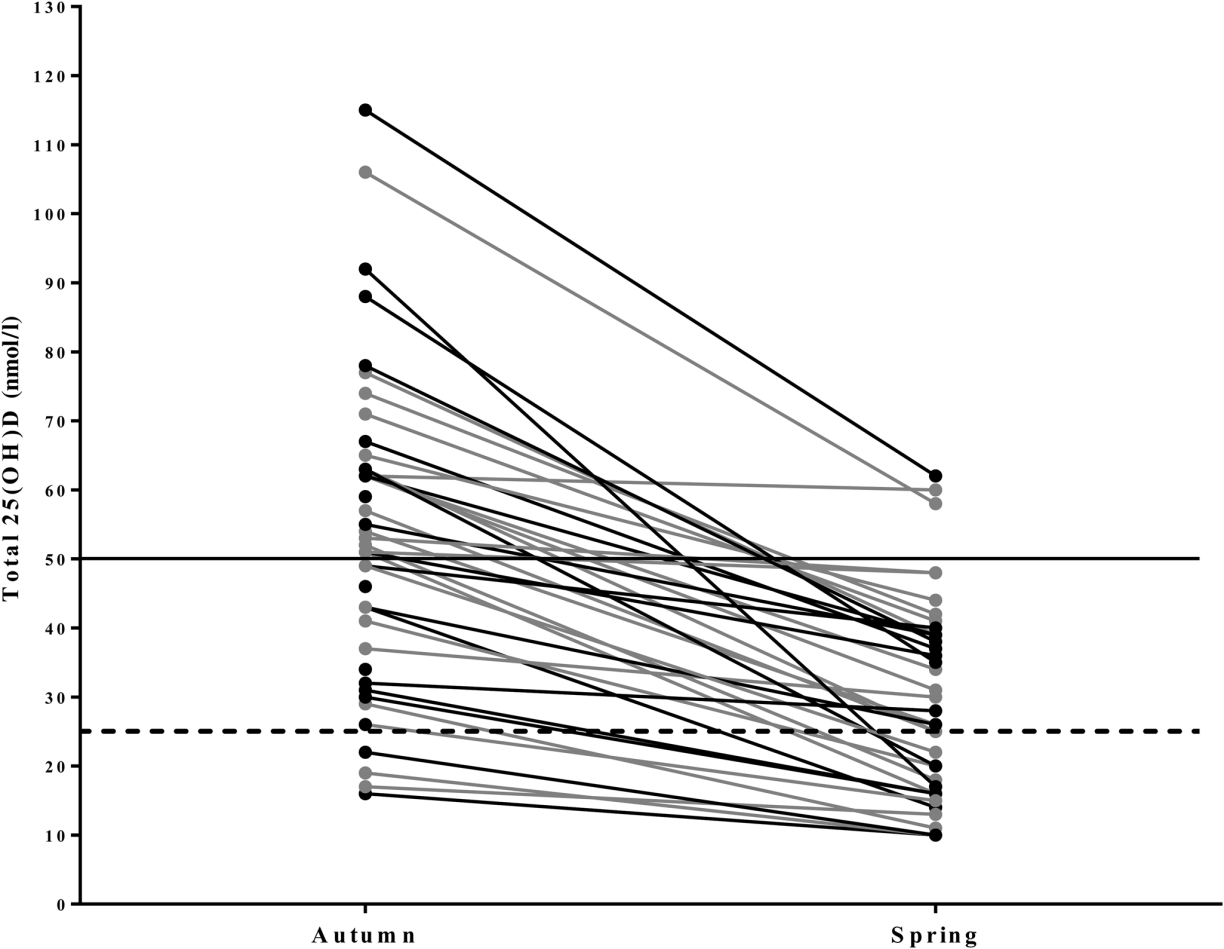
Line graph illustrating the individual variation of 25-hydroxyvitamin D (25(OH)D) concentrations in university-level athletes assessed in autumn (*n* 47) and spring (*n* 47). 25(OH)D statuses of <25 nmol/l are considered deficient (----). 25(OH)D statuses of >50 nmol/l are considered sufficient (–––). ●, Indoor athletes; ●, outdoor athletes.

**Fig. 3.**
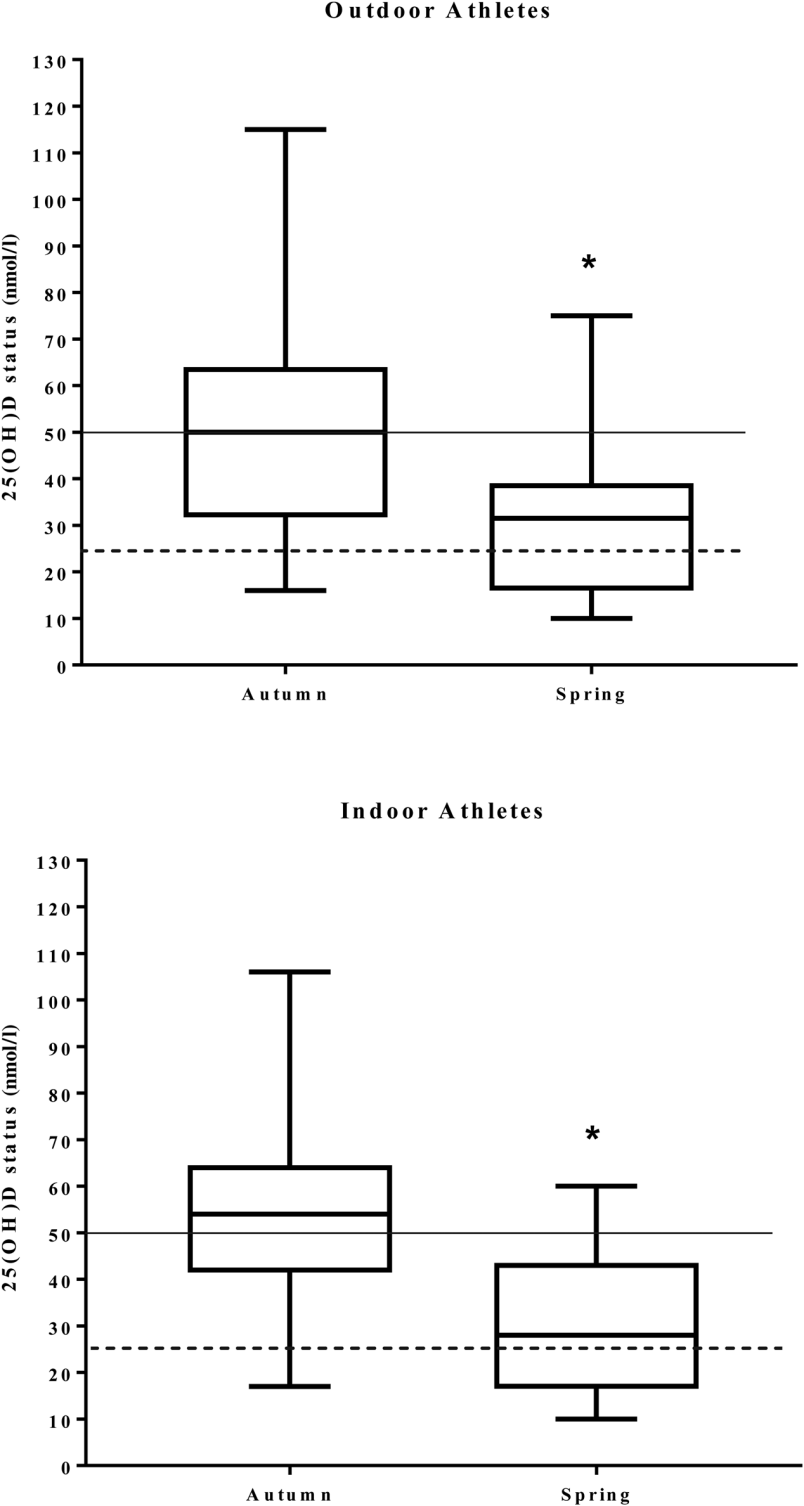
Box plots illustrating the distribution of 25-hydroxyvitamin D (25(OH)D) concentrations in outdoor and indoor university-level athletes throughout a competitive sporting season. The central vertical lines in the box plots indicate mean values of participants during autumn and spring. 25(OH)D statuses of <25 nmol/l are considered deficient (----). 25(OH)D statuses of >50 nmol/l are considered sufficient (––––). * Significantly different between seasons (*P* < 0·05).

Serum vitamin D status for the entire population decreased from 56·1 (sd 23·3) nmol/l in autumn to 31·0 (sd 16·5) nmol/l in spring (*P* < 0·001) (Figs 2 and 3). During autumn, 43 % (*n* 20) of the university athletes had an insufficient (≤50 nmol/l) vitamin D status, with 11 % classified as deficient (≤25 nmol/l; *n* 5) according to the UK current guidelines^(24)^ in autumn. Vitamin D status further decreased in the course of our study, with 79 % (*n* 37) of participants insufficient and 34 % (*n* 16) deficient at the spring measurement. Moreover, six of our athletes had a ‘severe deficiency’ status (<12·5 nmol/l^(24)^). Training and competing indoors did not result in significantly different autumnal 25-hydroxyvitamin D status when compared with outdoor athletes (57·7 (sd 22·1) *v.* 54·3 (sd 25·3) nmol/l, respectively; *P* = 0·589), nor did the spring measurement (31·0 (sd 16·1) *v.* 31·0 (sd 17·5) nmol/l; *P* = 0·994). PTH values are reported in Table 1. There was a significant negative association between PTH and vitamin D status in the combined groups and indoor groups. A significant negative association was only observed in the outdoor group during the spring term; all other associations are presented in Table 3.

**Table 3.** Correlations between vitamin D status, intake, parathyroid hormone (PTH) and performance parameters

	Autumn				Spring			
	Outdoor		Indoor		Outdoor		Indoor	
	*r*	*P*	*r*	*P*	*r*	*P*	*r*	*P*
Vitamin D intake (μg/d)*†	−0·054	0·827	−0·71	0·772	−0·356	0·489	0·194	0·591
PTH (pmol/l)*†	−0·215	0·314	−0·423	0·016	−0·577	0·012	−0·527	0·008
Knee extensor strength (Nm)‡	0·103	0·684	0·467	0·014	0·293	0·382	0·182	0·442
Handgrip (kg)‡	0·243	0·330	0·219	0·272	0·513	0·088	0·488	0·025
CMJ (cm)*‡	0·544	0·024	0·033	0·879	0·439	0·469	0·092	0·724
VO_2max_ (ml/kg per min)*‡	0·563	0·019	0·225	0·314	0·294	0·632	0·231	0·372

CMJ, counter movement jump; VO_2max_, aerobic fitness.

* Not normally distributed in autumn.

† Not normally distributed in spring.

‡ Partial correlations controlling for height, weight and BMI.

When divided into groups according to self-reported ethnicity, the Asian–Indian athletes were borderline deficient in the autumn (27·5 (sd 11·8) nmol/l), which declined further to severe serum 25-hydroxyvitamin D deficiency in the spring (12·8 (sd 3·9)  nmol/l). This was also reflected within the Asian-Chinese cohort during the autumn and spring (22·5 (sd 4·9) and 12·5 (sd 3·5) nmol/l, respectively). Although direct comparisons could not be made due to small population groups, the vitamin D levels of the White-Caucasian group were higher in autumn and spring (61·2 (sd 21·0) and 35·3 (sd 15·3) nmol/l, respectively).

### Vitamin D cut-off analysis

Results showed that vitamin D status had a significant effect upon lower body muscular strength, presented in Table 4. *Post hoc* tests showed that subjects who had an ‘above sufficiency’ (>75 nmol/l) vitamin D status during the autumn term presented with superior knee extension strength when compared with vitamin D-insufficient (25–50 nmol/l) subjects (317·3 (sd 114·2) *v.* 227·8 (sd 61·4) Nm, respectively; *P* = 0·019). However, no other vitamin D status categories were statistically related to predictors of athletic performance or bone health during both seasons, which may be due to the small subject numbers in the different categories.

**Table 4.** Categories of vitamin D status across both seasons (Mean values and standard deviations)

	Autumn								Spring							
	Deficient (<25 nmol/l) (*n* 5)		Insufficient (25–50 nmol/l) (*n* 16)		Sufficient (50–75 nmol/l) (*n* 20)		Above sufficiency (>75 nmol/l) (*n* 6)		Deficient (<25 nmol/l) (*n* 16)		Insufficient (25–50 nmol/l) (*n* 21)		Sufficient (50–75 nmol/l) (*n* 4)		Above sufficiency (>75 nmol/l) (*n* 1)	
Physical performance	Mean	sd	Mean	sd	Mean	sd	Mean	sd	Mean	sd	Mean	sd	Mean	sd	Mean	sd
Handgrip (kg)	34·3	10·1	39·4	12·5	39·9	8·9	39·4	11·9	35·3	10·1	41·5	12·4	36·4	10·6	34·0	0
Knee extensor strength (Nm)	236·9	32·0	227·8	61·4	243·6	73·2	317·3*	114·2	238·9	77·3	260·0	70·4	238·8	47·0	226·6	0
CMJ (cm)†	31·8	13·7	28·2	10·1	30·0	5·6	34·5	10·3	32·0	8·7	32·8	7·2	36·7	8·4	29·6	0
VO_2max_ (ml/kg per min)†	41·1	1·2	41·9	2·0	43·9	2·9	43·9	3·9	42·8	3·6	43·1	2·5	43·5	2·9	45·1	0
4 % Radius																
BMC (g/cm)	1·26	0·32	1·39	0·35	1·46	0·34	1·39	0·19	1·30	0·31	1·47	0·32	1·28	0·30	1·3	0
Total CSA (mm^2^)	365·06	55·6	392·56	97·06	414·60	93·92	396·83	74·29	370·2	81·0	417·5	94·76	393·00	65·47	422·50	0
BMD (mm^3^)	343·86	56·43	369·71	70·82	354·94	47·17	357·50	62·46	356·12	65·75	362·1	54·17	322·84	27·74	308·09	0
Trab vBMD (mg/cm^3^)†	251·92	81·10	239·79	55·62	229·0	57·63	238·48	45·24	235·30	57·84	231·56	56·07	214·40	21·78	170·45	0
66 % Radius																
BMC (g/cm)†‡	1·63	1·29	1·44	0·60	1·55	0·78	1·44	0·64	1·49	0·86	1·61	0·77	1·26	0·15	0·92	0
Total CSA (mm^2^)†‡	281·13	266·50	229·14	140·43	265·45	177·00	462·08	547·5	247·52	184·25	275·00	172·95	555·94	673·15	147·75	0
BMD (mm^3^)	644·32	125·1	675·20	106·64	643·43	76·47	523·15	266·88	652·37	109·16	600·51	152·72	659·58	73·20	625·28	0
Crt CSA (mm^2^)†‡	99·56	48·17	73·99	29·21	84·58	23·49	71·08	32·61	100·13	45·50	105·27	51·29	80·43	3·69	65·00	0
Crt vBMD (mg/cm^3^)†‡	1075·35	44·10	1064·54	68·25	1080·09	36·47	1059·45	59·56	1057·74	70·86	1073·57	34·45	1062·75	60·56	1106·67	0
SSI (mm^2^)†‡	783·17	1134·98	429·38	370·81	448·03	548·14	529·23	612·36	542·18	642·64	461·10	524·92	622·97	768·66	240·27	0

CMJ, counter movement jump; VO_2max_, aerobic fitness; BMC, bone mineral content; CSA, cross-sectional area; BMD, bone mineral density; Trab, trabecular; vBMD, volumetric bone mineral density; Crt, cortical; SSI, strength strain index.

* *P* < 0·05 (one-way ANOVA between the ‘insufficient’ and ‘above sufficient’ groups).

† Not normally distributed in autumn.

‡ Not normally distributed in spring.

## Discussion

The aim of the present study was to investigate vitamin D status in UK university-level athletes and whether this was related to markers of performance. We found that vitamin D status decreased significantly from autumn to spring and that there were no differences between outdoor and indoor athletes (Table 1). Vitamin D intake was also minimal during the autumn (2·9 µg/d) and spring (3·2 µg/d). Seasons did not have an effect upon predictors of upper and lower body strength-related measurements, power and AF. We observed associations between vitamin D status, aspects of physical performance and AF; this was, however, only observed within the outdoor group. Granted, a limitation to this study is the use of the Yo-Yo intermittent recovery test to predict AF. However, the athletes recruited predominantly participated in team sports; therefore, the researchers regarded it more applicable to utilise this method due to the intermittent nature of their sports. Therefore, these findings of a significant correlation between vitamin D status and AF only within the outdoor athletes could be attributed to the majority of outdoor athletes partaking in team sports (such as rugby, lacrosse, etc.). Thus, these athletes may have scored higher in the Yo-Yo intermittent recovery test contributing to higher AF results due to this potential advantage.

Insufficient vitamin D status is common within Europe^(15)^, especially within the professional athletic community^(1,2,10)^. Reduced vitamin D availability can have serious health implications to bone and muscular function^(3)^. There are adequate data regarding the vitamin D status of university athletes at varying latitudes within the USA^(17,18,26,27)^ but little evidence regarding the UK. To our knowledge, this is the first study to determine the relationship between seasonal vitamin D status, bone health and exercise performance within UK-dwelling university athletes.

Our results show that UK athletes are at risk of vitamin D deficiency (<25 nmol/l), as 43 % of our participants were deficient in spring and 79 % were classified as insufficient (<50 nmol/l). This prevalence of vitamin D deficiency/insufficiency far exceeds that reported in the USA^(28)^. We observed a significant decline in vitamin D status from autumn to spring in agreement with the findings of Peeling *et al.*^(16)^. However, vitamin D status was consistently higher in their Australia-based athletes: 122, 105 and 75 nmol/l in autumn, spring and winter, respectively, compared with our cohort. Similarly, others have reported higher vitamin D status in US (122 nmol/l^(17)^ and 100·1 nmol/l^(18)^), Greek (86–118 nmol/l)^(19)^ and Irish (76·5 nmol/l)^(29)^ athletes. These higher statuses could be attributed to the geographical latitudes of these four studies: 41°N^(16)^, 34°N^(18)^, 31°S^(16)^ and 36°N^(19)^. Converse to our findings, a higher baseline vitamin D status within the Irish cohort is probably due to almost 25 % of their athletes reporting that they take vitamin D supplements. Moreover, forty-three of their athletes reported equatorial travel and a further fifteen reported the regular use of sunbeds, which play a vital role in the synthesis of pre-vitamin D_3_^(13)^.

Ethnicity also played a significant role in the prevalence of vitamin D status within our athlete cohort; those that were of Asian–Chinese or Asian–Indian descent presented as either vitamin D deficient (<25 nmol/l) or severely deficient (<12·5 nmol/l) during both seasons. This was reflected within a large cross-sectional study investigating the vitamin D levels of 342 professional football players in Qatar^(30)^. It was found that vitamin D status was lower within their Middle-Eastern (53·9 nmol/l), Persian (42·9 nmol/l) and Gulf countries (45·7 nmol/l) athletes when contrasted to their white-Caucasian group (87·6 nmol/l). However, these vitamin D levels were considerably higher than our cohort as this was measured in the summer term only. Hamilton *et al.*^(30)^ also measured the lower body muscular strength of their footballers utilising isokinetic dynamometry; they also evaluated their absolute peak torque against vitamin D clinically significant cut-offs for vitamin D status. They observed that non-dominant leg hamstring concentric 300°/s and eccentric 60°/s strength were significantly greater in the 20–30 (50–75 nmol/l) *v.* the <10 ng/ml (<25 nmol/l) group. However, these findings were not consistent across all measurements of the dominant concentric/eccentric predictors of lower body muscle strength. In our study we did not find a consistent effect of vitamin D status categories on markers of muscular strength; however, this may have been due to our small subject number. Further research should focus upon these clinical categories when examining the associations between vitamin D and physical performance.

Despite the significant decline in vitamin D status from autumn to spring, vitamin D intakes did not differ (2·7 (sd 2·3) and 3·2 (sd 3·3) µg/d, respectively) across the seasons and are representative of a UK population^(31)^. As the data were collected in the spring of 2016, this provides a novel insight into the vitamin D intake of a young adult cohort before vitamin D made popular headlines; the results thus support the changes issued by the Department of Health for a higher recommended intake for adults^(24)^. Therefore, inadequate UVB exposure during wintertime and an unbalanced diet could be the driving force for a borderline deficient vitamin D status of 31 nmol/l in spring. Our dietary intake results are low in contrast to other studies which reported higher vitamin D statuses^(17)^. These were, however, likely to be the result of food fortification (e.g. milk and cereal) programmes^(28)^. Ca intake fell below current recommendations of 700 mg/d^(25)^ within the indoor group during the spring (678 mg/d). However, it is unlikely that this could be a contributing factor to a significantly lower proximal bone mineral density and trabecular bone mineral density observed in the indoor group during the spring *v.* the outdoor group (381·2 (sd 53·9) *v.* 339·4 (sd 53·5) mm^3^). As this study did not measure dietary intake utilising a FFQ the authors were unable to account for the decline in intake and determine which Ca food sources were potentially omitted. PTH was also notably higher in the indoor group during the spring (7·0 (sd 5·1) *v.* 5·5 (sd 2·2) pmol/l). Despite similar mean 25-hydroxyvitamin D status within the spring term, this could be attributed to a higher proportion of the indoor athletes presenting with deficiency (<25 nmol/l; *n* 11; 44 %) when contrasted to the outdoor group during the spring (*n* 6; 27 %). However, as we did not analyse bone turnover markers within this investigation such as carboxyl terminal pro-peptide (a biochemical marker of bone formation) or c-terminal cross-linking telopeptides (a biochemical marker of bone resorption) it is difficult to determine whether this could be the cause for a higher PTH in the indoor group.

This observational study demonstrated that there is a potential relationship between vitamin D and predictors of physical performance. This is reflected by the findings in autumn for knee extensor strength (*r* 0·378) and AF (*r* 0·391) and is supported by previous literature^(32)^. Despite the significant decline in vitamin D status across the seasons, handgrip strength (*r* 0·385) was the only measurement to be associated with vitamin D. These findings could be attributed to a lack of control in training programmes as this is challenging to do so within this group as their training is individual determined, which is reflected in previous research for university athletes^(17,18,27)^. However, our findings are in accordance with other studies regarding the relationship between vitamin D and physical performance^(33)^. Furthermore, muscular strength did not change across the seasons and concurs with findings from Close *et al*.^(21)^. Parallel to this, there was no improvement in physical parameters following supplementation in Irish university Gaelic footballers^(29)^ with a lower mean vitamin D status than our baseline and previous authors at 47·4 and 43·1 nmol/l for their intervention and placebo groups, respectively. Although we did not supplement our cohort with vitamin D, it is still comparative for baseline measurements, specifically as both aforementioned were the only UK-based cohorts to our knowledge in this young adult population.

### Conclusions

In conclusion, we provide clear data to show that non-supplemented UK university athletes are at significantly increased risk of vitamin D deficiency, with prevalence of the ‘deficiency’ threshold for vitamin D recently set by the Scientific Advisory Committee on Nutrition (SACN) being 7 % in the autumn and 34 % in spring. To our knowledge, this is the first longitudinal observational study on UK university athletes. This thereby highlights the need of the importance of an adequate diet, sunlight exposure and the potential requirement for wintertime supplementation in the prevention of vitamin D deficiency in young adults. Our findings of a positive link between an adequate vitamin D status and physical performance during the autumn term is of interest and certainly warrants further investigation.

## References

[1] Farrokhyar F, Tabasinejad R, Dao D, (2015) Prevalence of vitamin D inadequacy in athletes: a systematic-review and meta-analysis. Sports Med 45, 365–378. 25277808 10.1007/s40279-014-0267-6

[2] Dahlquist DT, Dieter BP & Koehle MS (2015) Plausible ergogenic effects of vitamin D on athletic performance and recovery. J Int Soc Sports Nutr 12, 33. 26288575 10.1186/s12970-015-0093-8 PMC4539891

[3] Girgis CM, Clifton-Bligh RJ, Hamrick MW, (2013) The roles of vitamin D in skeletal muscle: form, function, and metabolism. Endocr Rev 34, 33–83. 23169676 10.1210/er.2012-1012

[4] Allison R, Farooq A, Hamilton B, (2015) No association between vitamin D deficiency and markers of bone health in athletes. Med Sci Sports Exercise 47, 782–788. 10.1249/MSS.0000000000000457 25058327

[5] Neal S, Sykes J, Rigby M, (2015) A review and clinical summary of vitamin D in regard to bone health and athletic performance. Phys Sportsmed 43, 161–168. 25797288 10.1080/00913847.2015.1020248

[6] Jastrzębska M, Kaczmarczyk M, Michalczyk M, (2018) Can supplementation of vitamin D improve aerobic capacity in well trained youth soccer players? J Hum Kinet 61, 63–72. 29599860 10.2478/hukin-2018-0033 PMC5873337

[7] Lewis RM, Redzic M & Thomas DT (2013) The effects of season-long vitamin D supplementation on collegiate swimmers and divers. Int J Sport Nutr Exerc Metab 23, 431–440. 23475128 10.1123/ijsnem.23.5.431 PMC4395005

[8] Wyon MA, Koutedakis Y, Wolman R, (2014) The influence of winter vitamin D supplementation on muscle function and injury occurrence in elite ballet dancers: a controlled study. J Sci Med Sport 17, 8–12. 23619160 10.1016/j.jsams.2013.03.007

[9] Farrokhyar F, Sivakumar G, Savage K, (2017) Effects of vitamin D supplementation on serum 25-hydroxyvitamin D concentrations and physical performance in athletes: a systematic review and meta-analysis of randomized controlled trials. Sports Med 47, 2323–2339. 28577257 10.1007/s40279-017-0749-4

[10] Owens DJ, Fraser WD & Close GL (2015) Vitamin D and the athlete: emerging insights. Eur J Sport Sci 15, 73–84. 25131312 10.1080/17461391.2014.944223

[11] Davey T, Lanham-New SA, Shaw AM, (2016) Low serum 25-hydroxyvitamin D is associated with increased risk of stress fracture during Royal Marine recruit training. Osteoporos Int 27, 171–179. 26159112 10.1007/s00198-015-3228-5

[12] Ruohola JP, Laaksi I, Ylikomi T, (2006) Association between serum 25(OH)D concentrations and bone stress fractures in Finnish young men. J Bone Miner Res 21, 1483–1488. 16939407 10.1359/jbmr.060607

[13] Wacker M & Holick MF (2013) Sunlight and vitamin D. Dermatoendocrinology 5, 51–108. 10.4161/derm.24494 PMC3897598 24494042

[14] Webb AR, Kline L & Holick MF (1988) Influence of season and latitude on the cutaneous synthesis of vitamin D_3_: exposure to winter sunlight in Boston and Edmonton will not promote vitamin D_3_ synthesis in human skin. J Clin Endocrinol Metab 67, 373–378. 2839537 10.1210/jcem-67-2-373

[15] Cashman KD, Dowling KG, Škrabáková Z, (2016) Vitamin D deficiency in Europe: pandemic? Am J Clin Nutr 103, 1033–1044. 26864360 10.3945/ajcn.115.120873 PMC5527850

[16] Peeling P, Fulton SK, Binnie M, (2013) Training environment and vitamin D status in athletes. Int J Sports Med 34, 248–252. 22972245 10.1055/s-0032-1321894

[17] Halliday T, Peterson N, Thomas J, (2011) Vitamin D status relative to diet, lifestyle, injury, and illness in college athletes. Med Sci Sports Exerc 43, 335–343. 20543748 10.1249/MSS.0b013e3181eb9d4d

[18] Villacis D, Yi A, Jahn R, (2014) Prevalence of abnormal vitamin D levels among Division I NCAA athletes. Sports Health 6, 340–347. 24982708 10.1177/1941738114524517 PMC4065560

[19] Koundourakis NE, Androulakis NE, Malliaraki N, (2014) Vitamin D and exercise performance in professional soccer players. PLOS ONE 9, e101659. 24992690 10.1371/journal.pone.0101659 PMC4081585

[20] Bescós García R & Rodríguez Guisado FA (2011) Low levels of vitamin D in professional basketball players after wintertime: relationship with dietary intake of vitamin D and calcium. Nutr Hosp 26, 945–951. 22072336 10.1590/S0212-16112011000500004

[21] Close GL, Leckey J, Patterson M, (2013) The effects of vitamin D(3) supplementation on serum total 25[OH]D concentration and physical performance: a randomised dose–response study. Br J Sports Med 47, 692–696. 23410885 10.1136/bjsports-2012-091735

[22] Owens DJ, Sharple AP, Polydorou I, (2015) A systems-based investigation into vitamin D and skeletal muscle repair, regeneration, and hypertrophy. Am J Physiol Endocrinol Metab 309, E1019–E1031. 26506852 10.1152/ajpendo.00375.2015

[23] Bangsbo J, Iaia FM & Krustrup P (2008) The Yo-Yo intermittent recovery test. Sports Med 38, 37–51. 18081366 10.2165/00007256-200838010-00004

[24] Scientific Advisory Committee on Nutrition and Department of Health (2016) Vitamin D and health. https://www.gov.uk/government/groups/scientific-advisory-committee-on-nutrition (accessed July 2019).

[25] British Nutrition Foundation (2017) Nutrition requirements – British Nutrition Foundation. https://www.nutrition.org.uk/nutritionscience/nutrients-food-and-ingredients/nutrient-requirements.html (accessed August 2019).

[26] Heller JE, Thomas JJ, Hollis BW, (2015) Relation between vitamin D status and body composition in collegiate athletes. Int J Sport Nutr Exerc Metab 25, 128–135. 25028792 10.1123/ijsnem.2013-0250

[27] Forney LA, Earnest CP, Henagan TM, (2014) Vitamin D status, body composition, and fitness measures in college-aged students. J Strength Cond Res 28, 814–824. 23897020 10.1519/JSC.0b013e3182a35ed0 PMC8796704

[28] Calvo MS, Whiting SJ & Barton CN (2004) Vitamin D fortification in the United States and Canada: current status and data needs. Am J Clin Nutr 80, 1710–1716. 10.1093/ajcn/80.6.1710S 15585792

[29] Todd JJ, McSorley EM, Pourshahidi LK, (2017) Vitamin D_3_ supplementation using an oral spray solution resolves deficiency but has no effect on VO_2_ max in Gaelic footballers: results from a randomised, double-blind, placebo-controlled trial. Eur J Nutr 56, 1577–1587. 27015912 10.1007/s00394-016-1202-4 PMC5486642

[30] Hamilton B, Whiteley R, Farooq A, (2014) Vitamin D concentration in 342 professional football players and association with lower limb isokinetic function. J Sci Med Sport 17, 139–143. 23623203 10.1016/j.jsams.2013.03.006

[31] Public Health England (2018) National Diet and Nutrition Survey Results from Years 7 and 8 (Combined) of the Rolling Programme (2014/15 to 2015/16). https://www.gov.uk/government/statistics/ndns-results-from-years-7-and-8-combined (accessed August 2019).

[32] Książek A, Zagrodna A, Dziubek W, (2016) 25(OH)D_3_ levels relative to muscle strength and maximum oxygen uptake in athletes. J Hum Kinet 50, 71–77. 28149343 10.1515/hukin-2015-0144 PMC5260642

[33] Fitzgerald JS, Peterson BJ, Warpeha JM, (2015) Association between vitamin D status and maximal-intensity exercise performance in junior and collegiate hockey players. J Strength Cond Res 29, 2513–2521. 26313575 10.1519/JSC.0000000000000887

